# Minority Affirmations and the Boundaries of the Nation: Evidence From Québec

**DOI:** 10.1177/00323217231223400

**Published:** 2024-01-20

**Authors:** Colin Scott, Antoine Bilodeau, Audrey Gagnon, Luc Turgeon

**Affiliations:** 1 Department of Political Science, Concordia University, Montreal, QC, Canada; 2 Center for Research on Extremism, University of Oslo, Oslo, Norway; 3 School of Political Studies, University of Ottawa, Ottawa, ON, Canada

**Keywords:** national membership, cultural boundaries, shared values, intergroup relations, Quebec

## Abstract

Cultural criteria, like language skills and values, are salient features of nationalism discourse, reflecting imagined boundaries that separate ingroup from outgroup member when thinking about the nation. Despite their salience, the relationship between cultural membership criteria and other civic (attainable) or ethnic (ascriptive) national boundaries, along with their implications for intergroup relations, is contested. Using surveys from *N* = 6448 majority group members in the Canadian province of Québec, we argue cultural boundaries are empirically distinct from civic and ethnic ones. Cultural and civic criteria are both prominent prerequisites for membership into the Québécois national community, but cultural criteria show markedly divergent relationships with outgroup attitudes. The results underline the importance of conceptualizing cultural boundaries as a distinct set of national membership criteria and question the construct validity of blended ethnocultural boundary measures or approaches that aggregate civic and cultural criteria together as equally “attainable” markers of national membership.

## Introduction

How do ingroup members conceptualize their national community, and how do their criteria for membership relate to outgroup attitudes and support for policies affirming minority interests? How the boundaries of the nation are defined in the minds of citizens have important repercussions for intergroup relations. Much of the research on this topic has compared the consequences of endorsing civic criteria, which provide a pathway to national membership that is attainable to all those who choose to identify as co-nationals and show respect for the nation’s institutions and laws, with ethnic criteria, which restrict national membership based on ascribed characteristics like ethnicity, ancestry, or place of birth (Ignatieff, 1994; Kohn, 1944; Simonsen, 2016; Verkuyten and Martinovic, 2015). The normative and empirical work on this topic generally finds that endorsing ethnic criteria for national membership, which are immutable and out of the reach of outgroup members, is associated with more negative attitudes toward immigrants, while endorsing civic criteria, which, on their face, are attainable to outgroup members, tends to be associated with more receptive immigration attitudes.

However, research and theory have moved beyond the civic-ethnic dichotomy, with studies of public opinion finding individuals often endorse an assortment of criteria when conceptualizing the boundaries of the nation (Bilodeau and Turgeon, 2021; Hjerm, 1998; Lindstam et al., 2021). This research reflects shifts in public discourse (e.g. Couture and St-Louis, 2022; Giasson et al., 2010) and the rise of civic integration requirements for migrants (Goodman, 2014; Joppke, 2017; Lindstam et al., 2021), which have drawn attention to a more ambiguous set of criteria used to define national membership; criteria that emphasize the national culture, including a common language and shared set of values (Bouchard, 2015; Kymlicka, 2001; Larin, 2020; Reijerse et al., 2013).

The treatment of these cultural boundaries of the nation, however, is contested. Cultural criteria have been seen as important components of an “ethnocultural” or “ethnosymbolic” boundary (Smith, 2009), which include immutable characteristics like ancestry and place of birth, alongside language skills. However, cultural markers, including language, are not immutable, ascriptive characteristics, but are learned and therefore attainable to outsiders and conceptually distinct from ethnic markers (Bilodeau and Turgeon, 2021; Simonsen, 2016; Wright, 2011: 838–839). Unlike civic markers, which, in Western societies, reflect a commitment to political ideals such as democratic politics, individual rights and liberties, and the rule of law, cultural markers of national membership do not just require adherence to a civic contract, but also imply conformity to certain shared cultural attributes including, for example, a common language, values, or traditions (Gagnon, 2022). On their face, cultural and civic boundaries may overlap in some respects. For instance, speaking of a participatory democratic culture or a tradition of advocating for group equality invoke individual beliefs and values. They are, however, distinct in that cultural boundaries are defined explicitly in relation to the practices of a specific, dominant group that is perceived to be the foundation of the nation, and therefore exert assimilationist pressures on members of other groups that seek to be recognized as equals within the wider national community. In public debates over immigration, integration, and minority accommodations, cultural markers have been championed by conservative and progressive nationalists alike (Couture and St-Louis, 2022). Cultural boundaries are evidently a contested concept, raising questions about the validity of composite measures that blend cultural criteria together with other civic or ethnic national membership criteria.

We argue that despite their relationship to both ascriptive and attainable membership criteria, cultural boundaries warrant unique consideration as a distinctive set of boundary markers that are endorsed by civic- and ethnic-minded nationalists alike. Despite the growing attention paid to cultural criteria that use language and values to discern national membership, there has been little direct assessment of the divergent and convergent validity of such cultural boundary measures alongside other national membership criteria (but see Reijerse et al., 2013). In part, this is because much of the empirical survey research has relied heavily on the well-known national membership items included in the International Social Survey Program (ISSP), which includes items on language use and religion, but does not measure broader cultural membership criteria that are prominent in contemporary nationalism discourse, like sharing common values. Moreover, while language is highly relevant to discussions of national membership, the utility of a shared religion in distinguishing national boundaries is questionable in secular societies.

Drawing on surveys from the Canadian province of Quebec, and operationalizing cultural boundaries in terms of a shared language and set of values to be consistent with local nationalism discourse (Bouchard, 2015; Couture and St-Louis, 2022), we examine how different civic, ethnic, and cultural conceptions of the nation relate to one another, and analyze the degree of convergence between these three sets of membership criteria and different social and political attitudes toward an assortment of minority groups, namely Indigenous peoples, English speakers (who are a linguistic minority in Quebec), immigrants, and Muslims. We argue that while different ethnic, civic, and cultural boundary measures are related, they are unique constructs that demonstrate empirically distinct relationships with outgroup attitudes. As such, they are best measured separately. The Quebec case is highly informative for understanding the construction of national boundaries and their consequences for intergroup relations. Within Canada, Quebec is a predominantly francophone province, home to a national community that enjoys strong majority status within the province, but is itself a minority nation within the larger Canadian federation. Over the past two decades, Quebec nationalism has emphasized cultural issues that include not only the preservation of the French language, but also the promotion of “Quebec values,” namely, secularism (see Bouchard, 2015). As a result, Quebec nationalism has been gripped by a crisis of inclusion, with constant debate over the accommodation of minority cultural practices and their implications for Quebec national identity sparking tensions over the necessary conditions for belonging in the Quebec national community (Bouchard and Taylor, 2008; Giasson et al., 2010). Such debates have created fissures in Quebec nationalism, pitting conservative nationalists against more inclusive and pluralist views of the nation (Couture and St-Louis, 2022). In this context, this article explores the relationship between different conceptualizations of the national community and their relationships with various social and political attitudes toward minority groups.

## Citizens’ Representations of the National Community

Research investigating the relationship between national identity and outgroup attitudes tends to examine two distinct features: national identification, or the strength of one’s attachment to the national community and its importance to one’s sense of self; and national boundaries, the different ways the national community is defined and the criteria for membership. What has become clear from this work is that a strong national identification does not automatically give rise to anti-outgroup sentiment (Bilodeau et al., 2021; Brewer, 1999; Reicher and Hopkins, 2001). Probing the normative criteria used by citizens to define the boundaries of membership in the national community can help clarify the consequences of national identification for intergroup relations (Theiss-Morse, 2009). Whether a pathway to national membership is attainable to outgroup members, based on civic criteria like respecting laws and institutions or simply feeling like a co-national, or whether membership is unattainable, based on criteria like ethnicity and place of birth plays an important role in shaping how national identity relates to attitudes toward newcomers (Pehrson et al., 2009). Generally, stronger endorsements of civic criteria for national membership are associated with more positive outgroup attitudes and trust in others, while the opposite is true for stronger endorsements of ethnic criteria (Bilodeau and Turgeon, 2021; Lindstam et al., 2021; Reijerse et al., 2013).

Despite the growing literature on the boundaries drawn around the national community in the minds of citizens, there is less agreement on how cultural boundaries ought to be treated within this framework. In line with growing public discourse that places cultural criteria including a shared language or set of values front-and-center in the public debate over national membership (e.g. Bouchard, 2015; Giasson et al., 2010; Kymlicka, 2001; Larin, 2020), researchers have sought to go beyond the civic-ethnic dichotomy and better understand whether cultural criteria operate as a distinct set of considerations used to discern membership in the national community (Gagnon, 2022; Lindstam et al., 2021; Reeskens and Hooghe, 2010; Reijerse et al., 2013). Early research and theorizing often grouped ethnic and cultural boundaries together in a single “ethnocultural” understanding of the nation (Kohn, 1944; Smith, 2009). However, the equation of cultural and ethnic factors has been challenged (Kymlicka, 2001; Shulman, 2002; Zimmer, 2003). For example, Kymlicka (1999) notes that cultural boundaries of the nation differ from ethnic markers as they remain in principle open to outsiders.

Cultural markers like language are ambiguous membership criteria. For instance, Hjerm (1998: 336–340) refers to the “dual nature of language,” with language operating as either an ethnic or a civic criterion, depending on the perspective. To some, language may serve as a partitioning line demarcating belonging to a common group, in which case the criterion is a marker of ethnic boundaries. For others, however, language use is instrumental to full participation in the democratic process and civil society, making language skills an important tool to engage in civic membership. Such ambiguity presents a serious challenge to the classification of cultural boundary markers when thinking about the criteria for membership in the national community. Cultural membership criteria are attainable for those who wish to become part of the collective, while ethnic membership criteria are fundamentally unobtainable characteristics, thus undermining the construct validity of blended ethnocultural boundary markers. Kymlicka (1999) specifically points to Quebec as an example of a brand of nationalism that has been labeled ethnic but is in fact cultural as it accepts and welcomes immigrants as full members of the national community, conditional on their participation in a common culture defined in relation to the Québécois majority group.

The few empirical studies that have directly engaged with cultural representations of the nation have yielded mixed results. However, research drawing on survey data from high school students in different European countries (Reeskens and Hooghe, 2010), as well as adults across Canada, including sizable samples from Quebec (Bilodeau and Turgeon, 2021), suggests cultural boundaries defined by language use overlap with civic boundaries, reflecting a common set of attainable national membership criteria. In other European samples, however, results support the opposite claim as ethnic and cultural membership criteria are found to be highly correlated and treated as a single ethnocultural factor (Lindstam et al., 2021). Others still find that cultural membership criteria are sufficiently distinct from other civic and ethnic criteria to warrant their own latent factor, which, like ethnic criteria, is associated with more negative outgroup attitudes (Reijerse et al., 2013).

These inconclusive findings may be attributed to different operationalizations of civic, ethnic, and cultural boundaries or the fact that national boundary definitions are imbued with contextual meaning and may not be directly comparable across contexts. Researchers have relied on contrasting operationalizations of cultural boundaries as either unique dimensions used to represent the national community (Gagnon, 2022; Reijerse et al., 2013), or as a blended set of criteria that treats cultural and civic criteria as equally attainable characteristics demarcating national membership (Bilodeau and Turgeon, 2021; Lindstam et al., 2021; Reeskens and Hooghe, 2010). As such, the convergent validity of cultural and civic boundary measures is an open empirical question. The fact that cultural markers including language and values can be learned and shift over time brings into question the construct validity of unidimensional “ethnocultural” membership criteria drawing on both attainable and unattainable boundary markers. Furthermore, as Reeskens and Hooghe (2010) observe in their cross-national analysis of the ISSP national boundary items, responses to the national boundary scales do not show adequate measurement invariance across countries. This suggests the measurement properties of the ISSP national boundary items differ sufficiently enough from one country to the next that the scale may not be measuring the same construct in each country. Alongside comparative public opinion research, we therefore also need more context-specific analyses of how national communities perceive the boundaries of their nations and the consequences of such perceptions for intergroup relations.

Civic and ethnic national boundaries are ideal types and in practice, individuals endorse an assortment of different civic, ethnic, and cultural criteria to discern membership in the national community (Hjerm, 1998; Lindstam et al., 2021). Using qualitative data from semi-structured interviews in Quebec, Gagnon (2022) explores how individuals distinguish different ethnic, civic, and cultural conceptions of the Québécois nation, and the implications these different understandings of the national community have for the way individuals discuss immigration and ethnocultural diversity in the province. As is the case in survey research, participants that most clearly endorsed civic and ethnic conceptions of the Quebec nation reported more positive and negative opinions about immigration and ethnocultural diversity, respectively. While individuals clearly distinguished civic and ethnic criteria, cultural conceptions of the nation were more ambiguous as these boundaries tended to be linked to either positive or negative opinions about diversity, depending on whether interviewees believed immigrants and ethnocultural minorities respect Quebec’s cultural distinctiveness.

Unlike civic or ethnic criteria, which are respectively open to all who “play by the rules” or closed entirely to those who lack certain traits, the relationship between strong endorsements of cultural boundaries and outgroup attitudes may be group specific, varying in part with individuals’ beliefs or stereotypes of different minority groups’ perceived ability or willingness to assimilate into the national culture. For example, while linguistic skills can certainly be acquired, some groups may be stereotyped as less inclined to acquire language skills or to use a language publicly. There may also be important differences in accent or dialect that are seen as demarcating group membership independent of language fluency. Likewise, emphasizing a certain interpretation of “shared national values,” such as an adherence to a particular understanding of secularism defined primarily in terms of the regulation of “ostentatious” religious symbols can create situations where some minority identities and practices are seen as less acceptable than others, and, by extension, incompatible with national membership. In such cases, strong assimilationist pressures, and the stereotype that certain groups will not (or cannot) assimilate, may foster exclusionary attitudes and lead to opposition to policies that affirm minority interests.

Such scenarios create challenges for the inclusion of outgroups into the national community when cultural boundaries of the nation are strongly pronounced. As such, we might expect representations of the national community that are heavily rooted in cultural criteria to be linked to negative attitudes toward minorities if certain minority groups are perceived as failing to adopt, or even of being incompatible with, the national culture. In such situations, we would anticipate observing a divergence in the relationship between civic and cultural national boundaries and outgroup attitudes toward more culturally distant groups. The public opinion literature, however, has had little to say on this issue in part because popular survey measures have generally limited their operationalization of cultural boundaries to the linguistic domain, blending them together with either civic or ethnic membership criteria, while failing to adequately measure core aspects of nationalist rhetoric rooted in shared values. The research has also focused primarily on immigration attitudes, raising questions about how national boundaries relate to support for other minority communities. More research is therefore needed to test the convergent and divergent validity of cultural boundary markers and their relationship not only to social evaluations of specific outgroups, but also on public support for policies that are affirm different minority groups. We know that minorities that are perceived as less committed to the national community are less likely to benefit from social solidarity and majority support (Banting et al., 2022; Harell et al., 2022; Scott, 2023). We believe research must also go beyond examining general social evaluations of outgroups, including anti-immigrant sentiment and generalized prejudice, to assess how different boundary definitions relate to one another and, in turn, to concrete policy proposals that affirm minority interests.

## The Present Research

This study investigates the relationships between civic, ethnic, and cultural conceptions of the nation, along with their associations with different outgroup attitudes and policy preferences affecting minorities. We use the province of Quebec as a relevant case study through which to examine the relationship between different national boundary measures, and their associations with an assortment of outgroup attitudes. We first examine response patterns to a modified version of the ISSP national membership scale that draws on key items from the ethnic and civic subscales along with an expanded measure of cultural membership criteria that explicitly taps criteria related to both language and values. We might expect a negative relationship between civic and ethnic criteria, given the conceptual distinction between attainable and ascribed attributes as well as the divergent relationships shown between civic and ethnic boundaries with inclusive attitudes. We acknowledge, however, that this relationship is nuanced, as other studies have observed strong national-level correlations between ethnic and civic factors, implying respondents that endorse ethnic boundaries also tend to adhere to support civic principles (Reeskens and Hooghe, 2010: 589). Concerning cultural criteria, our expectations are less clear. Given the centrality of language and values in public debates over identity and inclusion in the Quebec context, we anticipate that just about all respondents will endorse cultural criteria to some extent, and so cultural boundaries should be positively correlated with both civic and ethnic criteria. Our analyses will therefore begin with an exploratory assessment of the inter-relationships between these different criteria for national membership.

After examining the relationships between respondents’ endorsements of different national membership criteria, we test specific hypotheses about how majority group members who strongly endorse civic, ethnic, or cultural criteria feel toward four sets of outgroups in Quebec: Indigenous peoples, the province’s English-speaking minority, immigrants, and Muslims. In addition to respondents’ evaluations of these minority groups, we assess public support for policies that affirm or oppose their group interests. Our survey therefore includes an adapted version of the ISSP membership items designed to better reflect cultural criteria, allowing us to examine the independent relationships between different membership boundaries across a range of social and political attitudes toward different minority groups. We anticipate that civic criteria will be associated with more positive feelings toward minority groups and more support for policies that affirm their interests (Hypothesis 1). However, endorsing strong ethnic membership criteria would suggest that outgroup members are inadmissible as potential co-nationals, making the ingroup–outgroup distinction clear. We therefore expect those that strongly endorse ethnic criteria for national membership to report more negative outgroup attitudes and opposition to minority-affirming policies (Hypothesis 2).

The relationships between cultural criteria and outgroup sentiments and policy preferences are less clear. It is possible that cultural criteria are generally seen as attainable but are more attainable to some groups than others. For example, minority English-speakers and immigrants might be particularly likely to be excluded from the Quebec national community due to their positioning as outsiders by virtue of their mother tongue and place of birth. Religious minorities such as Muslims may also be viewed as outsiders because Quebec’s media landscape places a disproportionate emphasis on cultural conflict stemming from religious symbols (Giasson et al., 2010). The positioning of Indigenous peoples is less clear. Although Indigenous peoples are recognized as hailing from distinct minority nations, members of these communities have ancestral claim to the territory upon which the Québécois national community is situated. Because of the different historical relationships between minority English speakers, immigrants, religious minorities, and Indigenous peoples with the Quebec national community, we anticipate that cultural boundaries have different associations with outgroup attitudes depending on the group in question. While we have no a priori expectation regarding whether endorsements of national boundaries demarcated by cultural considerations around language and values relate to positive evaluations and support for Indigenous peoples, we do anticipate cultural boundaries to be especially powerful predictors of negative attitudes and opposition to policies affirming minority English speakers, immigrants, and Muslims (Hypothesis 3).

## Data and Procedure

Data to test these expectations are derived from a unique online survey fielded in Quebec between 2019 and 2020.^1^ The present analyses are restricted to members of the national majority group—white, Canadian-born Francophones residing in Quebec and who do not identify as Indigenous peoples. Respondents with missing values on the variables analyzed were excluded, leaving a final sample of *N* = 6448 majority group respondents from 277 different regions^2^ of the province. All respondents completed the survey in French. About half of the respondents (*n* = 3300; 51%) were women. Most had completed college or trade school (*n* = 2560; 40%), attended some university (*n* = 940; 15%), or had completed either a bachelor’s (*n* = 1252; 19%) or graduate degree (*n* = 315; 5%). The remainder (*n* = 1381; 21%) reported a high school education or less. Most respondents made between $30,000 and $60,000 per year (*n* = 1899; 29%) or between $60,000 and $90,000 (*n* = 1470; 23%), with fewer reporting lower incomes under $30,000 (*n* = 935; 14.1%) or higher incomes between $90,000 and $120,000 (*n* = 898; 14%) or over $120,000 (*n* = 1066; 17%).

### Outcome Measures

Two sets of outcome measures are examined in the study, reflecting different social and political attitudes. These questions assessed explicit prejudice toward different minority groups, and public opinion toward four policy proposals on issues that affect them. The groups were Indigenous peoples, minority English-speakers, immigrants, and Muslims. Explicit prejudice toward each of these four groups is measured using feeling thermometers, which asked respondents to rate each group on a scale from 0 to 10, where a score of 10 reflects very positive feelings and 0 reflects very negative feelings. Scores were recoded to range from 0 to 1, with higher scores reflecting more positive feelings toward the group. Prejudice toward immigrants was captured by combining evaluations of three different immigrant groups that are prominent in Quebec: Italians, Haitians, and Chinese.^3^ Respondents were then asked, “Using the same scale, how do you feel about the following groups in Quebec?” Their reported feelings toward “Anglophones,” “Indigenous peoples,” and “Muslims” were recoded to the same 0 to 1 scale.

Our second set of dependent variables capture support for concrete policy proposals that directly affect the interests of these minority groups. To measure policy preferences on issues affecting Indigenous peoples, English-speaking minorities, and immigrants, respondents were asked, “For you personally, do the following statements represent a very good idea, a good idea, a bad idea or a very bad idea?” To measure support for Indigenous peoples, respondents indicated whether it was a good idea “that Indigenous peoples have more control over the natural resources in their territories.” To measure support for minority English speakers, respondents indicated whether it was a good idea “to provide services in English to the English-speaking minority in Quebec.” Responses to both questions were dichotomized such that those saying the policy proposal is a “good” or “very good” idea are scored 1 and those thinking the proposal is a “bad” or “very bad” idea are scored as 0.^4^ To measure support for immigration, respondents were asked, “Do you think that the number of immigrants admitted each year to Quebec should increase, decrease, or remain more or less the same?” Responses to this question were recoded such that those preferring a decrease in the number of immigrants admitted each year to Quebec are scored 1 and those who think the number should increase or remain the same are scored as 0. Finally, two items measured support for banning religious symbols. In the first wave of the survey, respondents were asked whether “we should prohibit the wearing of religious symbols by state employees with coercive power (judges, police officers, and prison guards)” and, whether “we should prohibit the wearing of religious symbols by teachers in public schools.”^5^ These items (*r* = 0.78, *p* < .001) were rated on four-point scales recoded to range from 0 to 1 and averaged into a single measure of support for banning religious symbols. Higher scores reflect more support for banning religious symbols.

### National Boundary Representations

To measure our key set of predictor variables, the criteria used to assess membership in the Québécois national community, we draw on an adaptation of the commonly used items from the *International Social Survey Program*. Each set of membership criteria was measured with two items, in line with previous research (Wright, 2011). Respondents were presented with the following instructions:

According to some, you need to have the following traits to be a true Quebecer. For others, they are not important. How important are they to you? Are they very important, somewhat important, not very important or not important at all?

Civic boundaries were measured with two items, “Respect Quebec institutions and laws” and “Feeling like a Quebecker” (*r* = 0.25, *p* < .001). Ethnic boundaries were measured with the items, “Be born in Quebec” and “Having French Canadian ancestry” (*r* = 0.69, *p* < .001). Finally, cultural boundaries were measured with the items, “Speak French” and “Adhering to Quebec values” (*r* = 0.39, *p* < .001). The importance of each boundary for discerning what makes a “true Quebecker” was rated on a four-point scale, recoded to range from 0 (not important at all) to 1 (very important).

### Control Variables

We control for various individual- and community-level variables, including realistic and symbolic threats, community diversity, and social orientations that are expected to influence outgroup attitudes and support for minorities (Riek et al., 2006; Schlüeter and Scheepers, 2010; Van der Linden et al., 2017). These covariates control for respondents’ sociodemographic variables, generalized trust, social solidarity, intergroup contact, and threat perceptions stemming from symbolic and realistic threats. The cross-sectional nature of our study notwithstanding, controlling for these covariates of outgroup attitudes should provide a more precise estimation of the unique associations of national boundary criteria with minority affirmations. In terms of sociodemographics, we control for respondents’ age in years, whether they are female (scored 1, if so), their income, and their level of education. To control for social orientations, two further covariates are included in the analyses: respondents’ generalized trust in others and their support for increased government spending to the poor. Generalized trust is measured with a single item asking respondents whether, “Generally speaking, would you say that most people can be trusted or that you can never be too careful when dealing with others.” Support for increased spending on the poor was assessed with a survey item asking respondents whether they agree or disagree, on a four-point scale that “Government should spend more to help the poor.” As a proxy for the frequency of contact with minority groups, respondents were asked, “In your daily life, how often do you have conversations with immigrants or members of an ethnic minority? Would you say a few times a week, a few times a month, a few times a year, or almost never?” Responses were recoded to range from 0 to 1, with higher scores reflecting more frequent conversations.

Because our focus is on national boundary definitions, and not national identity strength, we also control for variation in respondents’ overall national identification with two survey items, each on a four-point scale, recoded to range from 0 to 1, and combined into a single measure with higher scores reflect a greater identification with Quebec.^6^ We also include a measure of the share of Indigenous peoples, English-speaking minorities, immigrants, and Arabs in respondents’ municipalities to control for contextual differences in potential exposure to minority groups. Finally, we control for several proxies for cultural and economic threats. At the individual level, we measure respondents’ symbolic threat with a single survey item measured on a 0 to 1 scale asking respondents, “Would you say you are very worried, somewhat worried, not very worried or not at all worried for the survival of the French language in Quebec?” To control for economic threat perceptions by asking their opinions on scales ranging from 0 to 10 about the future economic situation economic situation of their household and their province.^7^ At the contextual level, we include the proportion of unemployed residents in respondents’ municipalities by matching 2016 Canadian census data collected at the Census Subdivision level based on the first three digits of respondents’ postal codes.

## Results

### The Criteria for Membership in the Quebec National Community

**Figure 1.**
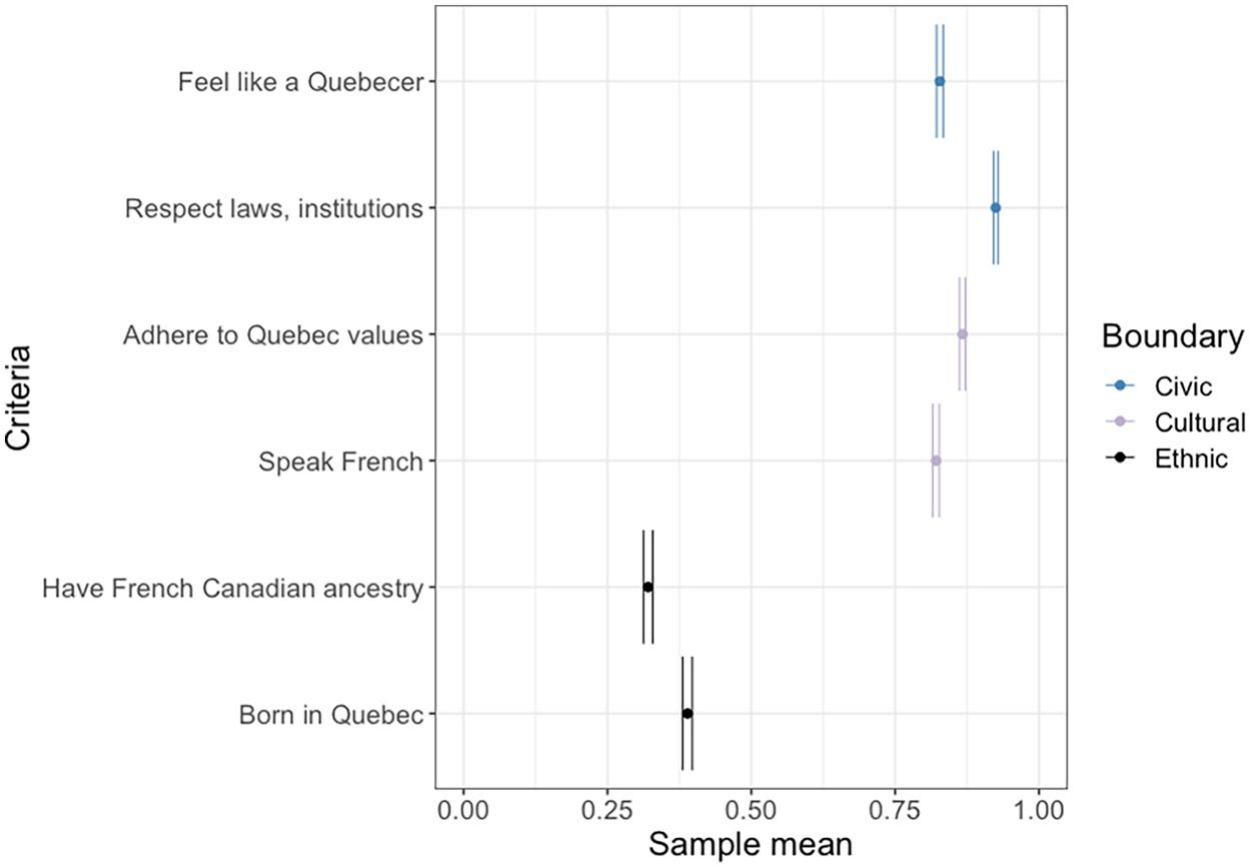
Average Endorsement of National Membership Criteria (95% Confidence Intervals Shown).

We begin our analysis with an assessment of how respondents describe the criteria used to discern membership in the Québécois national community. Respondents typically reported high levels of national identification with Quebec (*M* = 0.85, *SD* = 0.21). There is somewhat more disagreement regarding *what it means* to be Québécois. Figure 1 summarizes respondents’ endorsements of different civic, cultural, and ethnic criteria for national membership. In terms of the boundaries majority group members draw around the nation, membership is open and attainable. Most respondents strongly endorsed civic (*M* = 0.88, *SD* = 0.16) and cultural (*M* = 0.84, *SD* = 0.19) criteria. Speaking French, adhering to national values, respecting Quebec laws and institutions, and one’s own feeling of being Québécois, are all important prerequisites, in the minds of majority group members, for national membership. Much less frequently endorsed are the ascriptive traits: having been born in Quebec and having French-Canadian ancestry (*M* = 0.35, *SD* = 0.30). Nonetheless, the proportion of respondents endorsing exclusionary, ethnic membership criteria is not trivial.^8^

As others have noted, “civic” and “ethnic” boundaries are ideal types (Lindstam et al., 2021), and it is not uncommon for respondents to endorse different criteria to convey the boundaries they draw around the national community (Bilodeau and Turgeon, 2021; Hjerm, 1998). Respondents in our study are no exception. Descriptive statistics and bivariate correlations between different national boundaries measures are presented in Table 1. The correlations between ethnic, civic, and cultural membership criteria are all positive and statistically significant with relationships varying in strength from a weak correlation between civic and ethnic boundaries, to a moderate correlation between cultural and ethnic criteria. There was also a stronger, but still modest, relationship between civic and cultural items. While correlated, a confirmatory factor analysis finds a measurement model that allows cultural criteria to load on both civic and ethnic factors is an excellent fit to the data (*CFI* = 0.992, *TLI* = 0.98, *RMSEA* = 0.04). Evidently, cultural criteria are endorsed by respondents with different views on the attainability of national membership.

**Table 1.** Bivariate (Spearman) Correlations, Sample Means, and Standard Deviations for National Identity Variables.

	Civic boundary	Ethnic boundary	Cultural boundary
Civic boundary	–		
Ethnic boundary	0.09***	–	
Cultural boundary	0.47***	0.25***	–
Mean (*SD*)	0.88 (0.16)	0.35 (0.30)	0.84 (0.19)
*N*	6448	6448	6448

SD: standard deviation.

*** *p* < .001.

### National Boundaries and Attitudes Toward Minorities

Given that cultural criteria are embraced by those endorsing both civic and ethnic boundaries, this first set of exploratory analyses raises doubts about the appropriateness of grouping cultural criteria together with civic criteria to form an underlying measure of attainable national membership criteria. That cultural boundaries are also endorsed by those perceiving national membership in civic and ethnic terms, suggests individuals may have different ideals in mind when thinking about these criteria (see Gagnon, 2022). We cannot, with the survey data at hand, test this proposition directly. We can, however, see whether the relationship between cultural boundaries and outgroup attitudes more closely resemble those of civic or ethnic understandings of the nation. If, all else considered, cultural boundaries show a similar pattern of relationships as ethnic criteria with different social and political attitudes toward minority groups, this would further suggest that civic and cultural criteria are not measuring the same underlying construct and instead reflect different pathways to national membership that may not be equally attainable to all outgroups. Still, at face value, a unidimensional “ethnocultural” boundary is also questionable because, as mentioned earlier, ethnic markers are immutable while cultural boundaries relating to language and values are learned and in flux. We further test independence of the associations between each national boundary criteria with outgroup attitudes, examining the convergence between cultural boundaries of the nation with traditional civic and ethnic boundaries.

As outcome measures, we have explicit prejudice, operationalized as feeling thermometer scores toward each minority group, along with opinions toward four policy proposals (dis)affirming different minorities in Quebec: giving Indigenous peoples greater control over the natural resources in their territories; supporting the provision of English-language services to Quebec’s minority English-speaking community; supporting a decrease in the level of immigration to Quebec; and, banning religious symbols among public sector workers in positions of authority. Each item is coded 1 if the respondent supports the policy proposal or otherwise 0, except for support for banning religious symbols, which is a scale computed by averaging two survey items (see above). The use of linear and generalized linear mixed-effects models allows us to take contextual variation into account through the clustering of individuals within localities (i.e. forward sorting areas) while controlling for municipal-level variation in the unemployment rate and the size of different outgroups in respondents’ communities.^9^

A preliminary look at the outcome measures finds that respondents generally view these minorities in positive terms, with the notable exception of Muslims for whom explicit prejudice is much more pronounced. Indigenous peoples (*M* = 0.70, *SD* = 0.25), English speakers (*M* = 0.67, *SD* = 0.24), and a composite measure of feelings toward individuals from Italian, Haitian, and Chinese immigrant backgrounds (*M* = 0.72, *SD* = 0.19) are all rated positively on the feeling thermometer scales. Muslims, however, are evaluated much less favorably (*M* = 0.50, *SD* = 0.29). When it comes to supporting different policy proposals, respondents generally agree with different initiatives that affirm minority interests. Most think it is a good idea to give Indigenous peoples greater control over resources in their territories (*M* = 0.76, *SD* = 0.43). Support is also high for offering English-language services to Quebec’s minority English-speaking community (*M* = 0.71, *SD* = 0.46), and most respondents generally oppose a decrease in the levels of immigration to Quebec (*M* = 0.31, *SD* = 0.46). There is, however, strong public support shown for banning religious symbols among public sector employees in positions of authority (*M* = 0.77, *SD* = 0.30).

**Figure 2.**
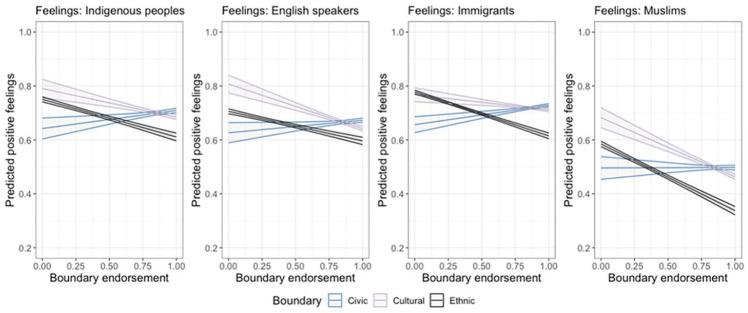
Predicted Positive Feelings Toward Minority Groups. Coefficients Estimated From Models in Table 2. 95% Confidence Intervals Shown.

First, we examine the independent relationships between civic, ethnic, and cultural boundaries and feeling thermometer scores toward each minority group. Results from the multivariate analysis are reported in Table 2. Interestingly, civic boundaries are not correlated with feelings toward Muslims. Otherwise, endorsing civic criteria such as feeling Québécois and respecting Quebec laws and institutions is generally associated with more positive feelings, at least toward three of the four groups. Endorsing ethnic criteria is associated with more negative feelings toward all four minority groups, controlling for other variables in the model. Our first hypothesis is therefore supported in as much as it relates to general feelings toward minorities, with the qualification that there is no independent relationship between endorsing civic criteria for national membership and feelings toward Muslims. Figure 2 visualizes the modeled independent relationships between each set of membership criteria and feelings toward different minority groups. As can be seen, the predicted relationships between cultural and ethnic criteria and different outgroup evaluations are negative, in contrast to the generally positive association between civic criteria and feelings about different outgroups.

**Table 2.** Multilevel Linear Mixed Effect Models Showing Effects of National Identity Variables on Feeling Thermometer Scores Toward Different Minority Groups. Standard Errors in Parentheses.

	(1)	(2)	(3)	(4)
	Indigenous peoples	English speakers	Immigrants	Muslims
Constant	0.47*** (0.03)	0.74*** (0.03)	0.50*** (0.02)	0.46*** (0.03)
Age	–0.00 (0.00)	0.00*** (0.00)	0.00** (0.00)	–0.00** (0.00)
Female	0.07*** (0.01)	0.05*** (0.01)	0.05*** (0.00)	0.05*** (0.01)
Education: College/CEGEP/Trade school	0.00 (0.01)	0.02* (0.01)	–0.00 (0.01)	0.01 (0.01)
Education: Some university	0.03* (0.01)	0.03** (0.01)	0.00 (0.01)	0.06*** (0.01)
Education: Bachelor degree	–0.00 (0.01)	0.02* (0.01)	–0.01 (0.01)	0.03** (0.01)
Education: Graduate degree	–0.01 (0.01)	–0.00 (0.01)	–0.02* (0.01)	0.03 (0.02)
Income: $30,000–$60,000	–0.02** (0.01)	0.00 (0.01)	0.01 (0.01)	0.00 (0.01)
Income: $60,000–$90,000	–0.03** (0.01)	0.02 (0.01)	0.00 (0.01)	–0.00 (0.01)
Income: $90,000–$120,000	–0.06*** (0.01)	0.01 (0.01)	0.00 (0.01)	–0.02 (0.01)
Income: $120,000 or more	–0.06*** (0.01)	0.01 (0.01)	–0.01 (0.01)	–0.02 (0.01)
Generalized trust	0.04*** (0.01)	0.04*** (0.01)	0.04*** (0.00)	0.10*** (0.01)
Supports government spending on poor	0.17*** (0.01)	0.04*** (0.01)	0.08*** (0.01)	0.15*** (0.01)
Frequent contact	0.06*** (0.01)	0.04*** (0.01)	0.05*** (0.01)	0.09*** (0.01)
Linguistic threat	–0.01 (0.01)	–0.21*** (0.01)	–0.05*** (0.01)	–0.13*** (0.01)
Economic situation: home	0.01*** (0.00)	0.01*** (0.00)	0.01*** (0.00)	0.01*** (0.00)
Economic situation: province	0.01*** (0.00)	0.01*** (0.00)	0.01*** (0.00)	0.01*** (0.00)
Economic situation: unemployment rate	–0.12 (0.17)	–0.29 (0.17)	–0.15 (0.14)	0.11 (0.20)
Proportion: indigenous	–0.53 (0.30)			
Proportion: English speakers		0.11*** (0.03)		
Proportion: immigrants			0.03 (0.02)	
Proportion: Arab				–0.13 (0.10)
National identification	0.09*** (0.02)	–0.02 (0.02)	0.07*** (0.01)	0.05* (0.02)
Civic boundaries	0.07** (0.02)	0.05* (0.02)	0.07*** (0.02)	0.00 (0.02)
Ethnic boundaries	–0.14*** (0.01)	–0.11*** (0.01)	–0.16*** (0.01)	–0.25*** (0.01)
Cultural boundaries	–0.11*** (0.02)	–0.17*** (0.02)	–0.06*** (0.02)	–0.22*** (0.02)
Num. Obs.	6448	6448	6448	6448
Localities	277	277	277	277
AIC	–557.50	–988.92	–4073.11	588.46

Standard errors in parentheses. Respondents are aggregated into localities based on the first three digits of their postal code. Contextual data at the municipality (i.e. census subdivision) level, compiled from the 2016 Canadian census. Reference categories for education and income are “high school or less” and “under $30,000,” respectively.

AIC: Akaike information criterion.

* *p* < .05; **p < .01; ***p < .001.

Cultural boundaries are associated with more negative evaluations of Indigenous peoples, English speakers, immigrants, and Muslims, all else equal. Although cultural boundaries are endorsed by those emphasizing both civic as well as ethnic representations of the nation (see Table 1), when it comes to their relationships with outgroup attitudes, like ethnic characteristics, those who strongly endorse cultural boundaries tend to also report less favorable outgroup attitudes. This negative relationship is especially strong between endorsements of cultural boundaries and negative sentiments toward linguistic and religious minorities. Civic and cultural membership criteria clearly show divergent correlations with measures of explicit prejudice toward different minority groups. These results question the construct validity of grouping cultural criteria together with civic criteria, as civic and cultural boundary endorsements have clearly different relationships with outgroup attitudes. This brings into question the degree to which cultural criteria for membership may truly be attainable in the minds of those who endorse national boundaries defined by cultural characteristics. The data also indicate that, while clearly related, cultural and ethnic boundaries demonstrate independent negative relationships that differ in the degree to which they are correlated with outgroup attitudes.

**Figure 3.**
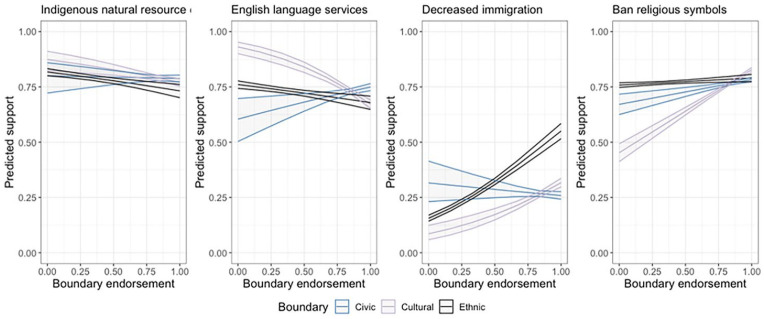
Predicted Support for Different Minority-Targeted Public Policies. Coefficients Estimated From Models in Table 3. 95% Confidence Intervals Shown.

Do these trends hold just for social evaluations about how much different minority groups are liked or disliked, or are membership criteria also associated with support or opposition for concrete policy proposals that target minority interests? How do those who draw different boundaries around the nation respond to policies that seek to support minority groups? The independent relationships between each set of membership criteria with policy preferences are presented in Figure 3 and multivariate results from generalized linear (Models 1–3) and linear (Model 4) mixed effect models are found in Table 3.

**Table 3.** Multilevel Generalized Linear (Models 1–3) and Linear (Model 4) Mixed Effect Models Showing Effects of National Identity Variables on Policy Supports, With Individual and Contextual Controls.

	(1)	(2)	(3)	(4)
	Indigenous resource control	Services to English-speaking minority	Decrease immigration	Ban religious symbols
Constant	–0.82** (0.31)	1.84*** (0.29)	–1.11*** (0.29)	0.30*** (0.03)
Age	–0.00 (0.00)	0.01*** (0.00)	–0.00 (0.00)	0.00*** (0.00)
Female	0.68*** (0.06)	–0.20*** (0.06)	–0.04 (0.06)	–0.09*** (0.01)
Education: College/CEGEP/Trade school	–0.07 (0.09)	0.12 (0.08)	–0.15* (0.08)	0.03** (0.01)
Education: Some university	0.24* (0.12)	0.44*** (0.11)	–0.84*** (0.11)	0.02 (0.01)
Education: Bachelor’s degree	–0.07 (0.10)	0.22* (0.10)	–0.52*** (0.10)	0.02 (0.01)
Education: Graduate degree	–0.08 (0.16)	0.24 (0.16)	–0.45** (0.17)	–0.04* (0.02)
Income: $30,000–$60,000	–0.36** (0.12)	–0.04 (0.10)	–0.43*** (0.09)	0.01 (0.01)
Income: $60,000–$90,000	–0.46*** (0.12)	–0.07 (0.10)	–0.33** (0.10)	0.02 (0.01)
Income: $90,000–$120,000	–0.60*** (0.13)	–0.27* (0.11)	–0.47*** (0.11)	0.02 (0.01)
Income: $120,000 or more	–0.96*** (0.13)	–0.31** (0.12)	–0.47*** (0.12)	0.01 (0.01)
Generalized trust	0.32*** (0.07)	0.27*** (0.07)	–0.62*** (0.07)	–0.03*** (0.01)
Supports government spending on poor	2.00*** (0.12)	0.90*** (0.12)	–0.83*** (0.12)	–0.08*** (0.01)
Frequent contact	0.35*** (0.09)	0.02 (0.08)	0.09 (0.09)	–0.04*** (0.01)
Linguistic threat	0.08 (0.13)	–1.61*** (0.13)	1.15*** (0.13)	0.13*** (0.02)
Economic situation: home	0.06** (0.02)	0.01 (0.02)	–0.06*** (0.02)	–0.01*** (0.00)
Economic situation: province	0.02 (0.02)	0.06*** (0.02)	–0.12*** (0.02)	0.00* (0.00)
Economic situation: unemployment rate	5.36** (1.84)	–0.67 (1.66)	–0.24 (1.90)	–0.43* (0.20)
Proportion: Indigenous peoples	–10.43*** (3.02)			
Proportion: English speakers		1.48*** (0.29)		
Proportion: Immigrants			0.43 (0.26)	
Proportion: Arab				0.23* (0.10)
National identification	0.90*** (0.19)	–0.10 (0.19)	0.07 (0.19)	0.08*** (0.02)
Civic boundaries	–0.07 (0.25)	0.67** (0.24)	–0.28 (0.25)	0.11*** (0.03)
Ethnic boundaries	–0.50*** (0.11)	–0.41*** (0.10)	1.89*** (0.11)	0.03** (0.01)
Cultural boundaries	–0.73** (0.23)	–1.88*** (0.23)	1.59*** (0.24)	0.38*** (0.02)
Num. Obs.	6448	6448	6448	6448
Localities	277	277	277	277
AIC	6399.58	7158.37	6697.17	1670.33
BIC	6562.09	7320.89	6859.68	1839.62

Standard errors in parentheses. Respondents are aggregated into localities based on the first three digits of their postal code. Contextual data at the municipality (i.e. census subdivision) level, compiled from the 2016 Canadian census. Reference categories for education and income are “high school or less” and “under $30,000,” respectively.

AIC: Akaike information criterion; BIC: Bayesian information criterion.

* *p* < 0.05; **p < 0.01; ***p < 0.001.

When it comes to policy preferences, civic national boundaries are not necessarily associated with increased support for minority groups. Those who more strongly endorse civic criteria as prerequisites of national membership also tend to have a higher likelihood of expressing support for English-language services for Quebec’s minority linguistic community. Independent of other variables included in the analysis civic boundaries is unrelated to support for indigenous control over natural resources and decreased immigration levels. It is also noteworthy that civic criteria are correlated with greater support for banning religious symbols from public sector workers. Previous survey research in Quebec observes that support for banning religious symbols is also found among those endorsing liberal values (Turgeon et al., 2019). Particularly given the salience of laws and institutions to regulate religious symbols in Quebec (see, for example, chapter 4, Bouchard, 2015), the data raise questions about the interpretation of certain “civic” prerequisites, and just what might be required of minorities to attain the standards of compliance with national laws and institutions (see also Larin, 2020). This further qualifies our expectations from the literature that civic criteria are typically tied to more pro-diversity attitudes. As our data suggest, there are important scope conditions around which civic endorsements may not be expected to relate to pro-diversity attitudes. In this case, our data suggest civic criteria may be mobilized to support bans on religious symbols and the reduction in generalized prejudice shown by those most strongly endorsing civic boundaries does not extend to evaluations of Muslims in the same way it does for different immigrant groups, minority English speakers, and Indigenous peoples.

With respect to the convergence in the relationships between ethnic and cultural boundaries and policy proposals on different diversity issues, we do not always see the same relationships with policy preferences as we did with general feelings toward different groups. Strong endorsements of ethnic criteria are tied to support for a decrease in immigration levels and banning religious symbols, and an opposition to greater Indigenous resource control and access to English-language services, controlling for the other factors considered here. This is as we would expect from the literature on ethnic nationalism and outgroup attitudes. We also see similar trends between cultural boundaries and minority-affirming policy preferences, as we do with ethnic boundaries. Here, however, it is noteworthy that we observe these unique associations between ethnic and cultural boundaries and both sets of outcome variables independent of each other. As Figures 2 and 3 make clear, the predicted levels of outgroup animosity and policy affirmation differ as a function of the degree of boundary endorsement. In our view, the results of these empirical analyses, together with a conceptual view of cultural boundaries being theoretically attainable while ethnic boundaries are innate and immutable, lead us to question the construct validity of blended ethnocultural boundary markers. In light of these findings, we encourage researchers to take stock of the unique relationships different civic, ethnic, and cultural boundaries of the nation have with social and political attitudes affecting minorities, rather than assuming a unidimensionality of any set of national membership criteria, whether ethnocultural or “attainable” boundaries that group civic and cultural criteria together.

## Discussion and Conclusions

Although civic and ethnic conceptions of the nation have been documented a great deal in the public opinion literature on national boundary definitions, the unique relationship between cultural criteria and outgroup attitudes has rarely been disentangled. In exploring the relationship between different civic, ethnic, and cultural conceptions of the Québécois national community, along with their associations with social and political attitudes toward minority groups in the province, this study makes several contributions to the literature on national boundary definitions, their associations with outgroup attitudes and support for minorities. We adapt the well-known ISSP national membership scale to better measure cultural considerations pertaining to shared values, in addition to language use. We believe this measurement approach offers an improvement on a narrower operationalization that relies primarily on language use, enhancing the face validity of our cultural boundaries measure in secular societies. Such an approach better reflect the contemporary debates surrounding nationalism and shared values (Gagnon and Larios, 2021; Giasson et al., 2010; Lægaard, 2007; Larin, 2020) and provides new empirical information on the relationship between culturally defined national boundaries and outgroup attitudes.

In line with other research (Bilodeau and Turgeon, 2021; Hjerm, 1998), the data clearly demonstrate how respondents perceive an assortment of criteria as necessary for membership in Quebec’s national community. Majority group members construct the boundaries around their nation by drawing heavily from civic and cultural boundaries that advance the official language, values, and institutions of the nation. Cultural boundaries, however, are a unique construct, empirically distinguishable from civic boundaries and endorsed by those favoring both civic and ethnic membership criteria. Cultural and civic boundaries generally have contrasting relationships with a range of minority-targeted issues. Here, our findings are more in line with those reported by Reijerse et al. (2013), who also adopt a broader operationalization of cultural boundaries beyond language use, and who document a negative relationship with pro-immigration attitudes. Taken together, our main results challenge the construct validity of conceptualizing civic and cultural boundaries as equally attainable pathways to national membership.

Our second contribution is to highlight the importance of group-level variation in understanding the relationship between different national boundaries and outgroup attitudes and policy preferences on minority-targeted issues. Both cultural and ethnic boundaries are associated with generalized prejudice, operationalized as negative feelings toward minority groups. Moreover, and quite strikingly, cultural boundaries, more so than ethnic boundaries, are consistently related to policy preferences that oppose minority interests, especially on highly symbolic issues. For example, those endorsing strong ethnic boundaries are in favor of a decrease in immigration, whereas cultural boundaries are independently related not only to anti-immigrant policy preferences, but also decreased support for the rights of linguistic and religious minorities in Quebec.

A notable exception to this trend, however, is that Indigenous peoples do not appear to face the same opposition by those strongly emphasizing cultural criteria for national membership. One potential explanation is that Indigenous peoples may be seen as belonging to another, separate minority nation and, as such, may be under less pressure to assimilate or conform to majority expectations. Considering their demographic weight, unlike immigrants and Anglophone Canadians, Indigenous peoples may also be perceived as less threatening to elements of Quebec culture, such as the French language or shared values. Here, however, we are confronted with an important limitation of our survey design. Without more fine-grained measures of the considerations individuals bring to mind in responding to our questions, we can only speculate on the reasoning behind why we do not observe a similar relationship when evaluating this proposal to facilitate indigenous self-determination. This is indeed an important limitation of the present research, one which we encourage future researchers to scrutinize in more detail.

As we expected, civic boundaries are most strongly endorsed by those showing lower levels of prejudice toward immigrants, Indigenous peoples, and linguistic minorities, but the correlation between civic boundaries and feelings toward Muslims is indistinguishable from zero. Indeed, civic boundaries are found to correlate positively with support for the banning of religious symbols from certain public servants. The association between civic boundaries and pro-diversity attitudes is apparently qualified, raising important questions about the broad inclusivity of civic boundaries and their consequences for the politics of minority claims-making. As others have noted (e.g. Larin, 2020), this empirical finding raises questions about the potential for civic criteria to be mobilized against minority interests in the name of protecting and promoting a majority-centric, assimilationist view of the nation that is closed to certain minorities.

A potential criticism of the present research is whether the findings generalize beyond the case of Québec. Comparative public opinion research using the ISSP’s national boundary scales has failed to establish measurement invariance in several European samples (Reeskens and Hooghe, 2010). Clearly, more research is needed into how specific national contexts influence the construction and representation of national membership boundaries. Still, we believe the Quebec case is an informative one for the broader study on national identity and intergroup relations. Our findings point to a positive relationship between ethnic and civic criteria, contrary to our expectations but in line with past work (see, for example, Reeskens and Hooghe, 2010). This may be explained by Quebec’s recent political context and the symbolic politics of religious identity in an explicitly secular state where, as in France and other societies, laws have been implemented to limit participation in select aspects of Quebec society to those expressing some form of religious identification (see Bouchard, 2015; Scott, 2007; Zubrzycki, 2016: ch. 9). Such an approach risks blurring the distinction between civic and cultural conceptions of the nation by legislating national values in ways that place conditions on the participation of select minority groups in society. Zimmer (2003) maintains that boundary making involves a dynamic process in which identity markers can be emphasized and used in different ways to (re)construct national identity. This process of reconstruction could explain why ethnic nationalists in the sample also endorse civic boundaries to some degree; if items measuring adherence to laws and institutions, such as bans on religious symbols, become embued with cultural meaning and are to be conceptualized as a mechanism to promote national heritage by targeting some aspects of minority cultural practices.

In recent years, with Quebec’s increasing tilt toward conservative nationalism (Couture and St-Louis, 2022), the symbolic politics of national values has been defined not only in terms of value incongruence with “les Anglais,” the province’s minority English-speaking communities, but also in terms of opposition to the religious minority communities that have settled in the province. Using ostensibly attainable criteria such as supporting national laws and institutions to exclude certain minority groups is a threat to the pluralist characterter of Quebec society. Again, we echo a concern that Larin (2020) describes as the “symbolic politics” of civic nationalism; where civic integration policies are used as a means of representing the national majority group at the expense of excluding certain minorities. In the face of an explosion of media coverage in Quebec over religious minority symbols and their supposed incompatibility with Quebec’s interpretation of secularism as a national value (e.g. Bouchard, 2015: ch. 4; Giasson et al., 2010; see also Scott, 2007), are considerations about ostensibly civic criteria like abiding by national laws and institutions inherently imbued with cultural significance? We believe our study of national boundary construction and their relationship to outgroup attitudes in Quebec uncovers some initial evidence to suggest that this may indeed be the case. Again, this presents an opportunity for further research to test these proposed explanations. It would be useful for future research to probe more clearly the considerations that respondents bring to mind when considering what values are important for national membership and why.

Our findings have important social and political implications for the extent to which national membership is attainable to all minorities. Often, integrationists and assimilationists alike will emphasize the attainable nature of national membership if newcomers would just embrace the cultural characteristics of the national community. Yet, our measures of majority group members’ endorsements of these cultural criteria showed the same negative relationship as ethnic representations of the nation with generalized outgroup prejudice. Indeed, on certain symbolic issues like the provision of English-language services to the minority Anglophone community and opposing bans on religious symbols, we observe that endorsements of strong cultural boundaries can act as an impediment to minority affirmations.

In contexts where the symbolic politics of national values has taken center stage, we believe more work is needed to untangle the relationship between cultural considerations and other attainable membership criteria. As it stands, however, we believe future research should treat these criteria as a separate dimension of national membership. As the data reported here illustrate, cultural boundaries are related to, but empirically distinct from, both civic and ethnic membership criteria. While attainable on face value, cultural boundaries show positive associations with exclusionary attitudes toward various minority groups but are nonetheless conceptually and empirically distinct from ethnic boundary markers because they are, at least in theory, learned markers of integration, and are independently related to policy preferences that oppose minority interests, especially on highly symbolic issues. Our study suggests cultural criteria may not be equally attainable to all. As cultural considerations make up a contested boundary of the nation, we suggest national membership criteria such as language and values be analyzed as a separate dimension to distinguish their relationships with outgroup attitudes from those of other ascriptive or attainable criteria.

## Supplemental Material

sj-docx-1-psx-10.1177_00323217231223400 – Supplemental material for Minority Affirmations and the Boundaries of the Nation: Evidence From Québec Supplemental material, sj-docx-1-psx-10.1177_00323217231223400 for Minority Affirmations and the Boundaries of the Nation: Evidence From Québec by Colin Scott, Antoine Bilodeau, Audrey Gagnon and Luc Turgeon in Political Studies

## Notes

1. All respondents were members of a Léger Marketing web panel.

2. Regions are defined with respect to the first three digits of respondents’ postal codes, corresponding to their “forward sorting area.” According to Statistics Canada (2017), Quebec has a total of 413 forward sorting areas. Although the survey design did not randomly sample based on this variable, respondents to our survey cover two-thirds of forward sorting areas across the province.

3. Respondents were asked, “Please tell us how you feel about Quebeckers of different origins, using a scale from 0 to 10, where 10 means that you have very positive feelings and 0 means that you have very negative feelings about Quebeckers of [Italian origin / Haitian origin / Chinese origin]”.

4. We opted for dichotomized dependent variables because we believe it is of substantive importance to examine overall (dis)agreement with these policy measures, regardless of the strength of the (dis)agreement. As a robustness check, we re-ran our analyses using an ordered logistic model, with outcome measures kept on their original scales. The results are robust to model specifications (see Appendix Table A1).

5. Between the two survey waves a policy change known as Bill 21 came into effect in Quebec which banned certain “ostentatious” religious symbols among public servants with coercive powers, including public school teachers. As a result, the language at the beginning of these two items in the second survey wave was changed to ask, “The government did well to prohibit ....” A bivariate linear regression with survey wave as the independent variable and support for banning religious symbols as the outcome finds that support was slightly lower for banning religious symbols in the second wave compared to the first wave, *b* = –0.04, *SE* = 0.01, *p* < .001. Multivariate results control for survey wave.

6. Respondents rated their agreement or disagreement with the following items: “The term ‘Quebecker’ describes me well”; and “Being a Quebecker is an important part of my identity” (*r* = 0.72, *p* < .001).

7. Specifically, respondents were asked, On a scale of 0 to 10, where 10 means it will be much better and 0 means it will be much worse, what do you think the financial or economic situation [in your home / in Quebec] will be in 10 years from now?

8. In the sample, 25% proportion of respondents indicated it was either “very important” or “somewhat important” to have French Canadian ancestry to be considered a true Québécois, while 34% agreed that it was “very” or “somewhat” important to have been born in Quebec. In the second wave of the survey, the questionnaire also asked respondents how important it is to “be White” to be a true Quebecker. Because the item was not included in the first survey wave, it is not included in the analyses presented here. Nine percent of second wave respondents did, however, agree that it was “somewhat” or “very” important to be ethnically white to be a member of the national community.

9. As a further robustness check, we re-run our analyses using linear or logistic regression analyses with clustered standard errors instead of mixed models. Results are robust to model specifications (see Appendix Tables A2 and A3).
