# Supplementary material for: Minority Affirmations and the Boundaries of the Nation: Evidence From Québec
Source: Polit Stud (Oxf). 2024 Jan 20;73(1):6–28. doi: 10.1177/00323217231223400 (PMC11802322; doi:10.1177/00323217231223400)
Supplement: sj-docx-1-psx-10.1177_00323217231223400 – Supplemental material for Minority Affirmations and the Boundaries of the Nation: Evidence From Québec [file sj-docx-1-psx-10.1177_00323217231223400.docx]

**Appendix A.** Supplementary materials

**Table A1**. Ordered logit models predicting attitudes toward indigenous natural resource control, provision of English-language services, and changing levels of immigration. Standard errors clustered at the forward sorting area in parentheses.

|  | (1) | (2) | (3) |
| --- | --- | --- | --- |
|  | Support for indigenous resource control | Support for English services | Support for decreased immigration |
| Age | -0.00* | 0.01*** | 0.00 |
|  | (0.00) | (0.00) | (0.00) |
| Female | 0.65*** | -0.19*** | 0.03 |
|  | (0.05) | (0.05) | (0.05) |
| Education: College/CEGEP/Trade school | -0.01 | 0.04 | -0.19** |
|  | (0.07) | (0.06) | (0.07) |
| Education: Some university | 0.29** | 0.23** | -0.76*** |
|  | (0.09) | (0.09) | (0.09) |
| Education: Bachelor degree | -0.05 | 0.11 | -0.55*** |
|  | (0.07) | (0.08) | (0.08) |
| Education: Graduate degree | -0.12 | 0.16 | -0.56*** |
|  | (0.11) | (0.13) | (0.13) |
| Income: $30,000 to $60,000 | -0.53*** | -0.18 | -0.39*** |
|  | (0.08) | (0.09) | (0.09) |
| Income: $60,000 to $90,000 | -0.46*** | -0.22* | -0.27** |
|  | (0.09) | (0.10) | (0.10) |
| Income: $90,000 to $120,000 | -0.62*** | -0.32*** | -0.35*** |
|  | (0.09) | (0.09) | (0.10) |
| Income: $120,000 or more | -0.85*** | -0.39*** | -0.43*** |
|  | (0.10) | (0.10) | (0.11) |
| Generalized trust | 0.30*** | 0.23** | -0.57*** |
|  | (0.05) | (0.06) | (0.05) |
| Supports government spending on poor | 2.29*** | 0.95*** | -1.06*** |
|  | (0.10) | (0.11) | (0.10) |
| Frequent contact | 0.32 | 0.12 | 0.08 |
|  | (0.08) | (0.07) | (0.08) |
| Linguistic threat | 0.12 | -1.53*** | 0.89*** |
|  | (0.11) | (0.11) | (0.11) |
| Economic situation: home | 0.06*** | 0.01 | -0.04** |
|  | (0.01) | (0.01) | (0.01) |
| Economic situation: province | -0.00 | 0.05*** | -0.11*** |
|  | (0.01) | (0.01) | (0.01) |
| Economic situation: unemployment rate | 3.19* | -0.58 | -1.55 |
|  | (1.34) | (1.18) | (1.50) |
| Proportion: indigenous | -9.90*** |  |  |
|  | (2.58) |  |  |
| Proportion: English speakers |  | 1.09*** |  |
|  |  | (0.18) |  |
| Proportion: immigrants |  |  | 0.27 |
|  |  |  | (0.20) |
| National identification | 0.71** | -0.22 | 0.22* |
|  | (0.17) | (0.17) | (0.16) |
| Civic boundaries | 0.01 | 0.66** | -0.10 |
|  | (0.19) | (0.20) | (0.20) |
| Ethnic boundaries | -0.57*** | -0.46*** | 1.75*** |
|  | (0.09) | (0.09) | (0.11) |
| Cultural boundaries | -0.67*** | -1.32*** | 1.62*** |
|  | (0.17) | (0.17) | (0.18) |
| Threshold 1 | -0.91*** | -3.26*** | -1.43*** |
|  | (0.23) | (0.24) | (0.24) |
| Threshold 2 | 0.70** | -1.57*** | 1.21*** |
|  | (0.23) | (0.24) | (0.24) |
| Threshold 3 | 3.10*** | 1.27*** |  |
|  | (0.23) | (0.23) |  |
| Num.Obs. | 6,448 | 6,448 | 6,448 |
| AIC | 14261.90 | 14028.23 | 11761.05 |

*Note:* * *p* < .05; ** *p* < .01; *** *p* < .001. Standard errors in parentheses, clustered by respondents’ forward sorting area (the first three digits of their postal code). Contextual data at the municipality (i.e., census subdivision) level, compiled from the 2016 Canadian census. Reference categories for education and income are “high school or less” and “under $30,000,” respectively. Dependent variables are ordered as follows: Support for indigenous resource control and support for the provision of English-language services to Quebec’s minority English-speaking communities are ordered categorical variables with four levels (from low to high: Very bad idea, bad idea, good idea, very good idea). Support for decreased immigration is an ordered categorical variable with three levels (from low to high: increase, stay the same, decrease).

**Table A2.** Ordinary least squares regression models showing effects of national identity variables on feeling thermometer scores toward different minority groups. Standard errors in parentheses, clustered by respondents’ forward sorting area.

|  | (1) | (2) | (3) | (4) |
| --- | --- | --- | --- | --- |
|  | Indigenous peoples | English speakers | Immigrants | Muslims |
| Constant | 0.47*** | 0.74*** | 0.50*** | 0.50*** |
|  | (0.03) | (0.03) | (0.02) | (0.02) |
| Age | -0.00 | 0.00*** | 0.00** | 0.00** |
|  | (0.00) | (0.00) | (0.00) | (0.00) |
| Female | 0.07*** | 0.05*** | 0.05*** | 0.05*** |
|  | (0.01) | (0.01) | (0.00) | (0.00) |
| Education: College/CEGEP/Trade school | 0.00 | 0.02* | -0.00 | -0.00 |
|  | (0.01) | (0.01) | (0.01) | (0.01) |
| Education: Some university | 0.03* | 0.03** | 0.00 | 0.00 |
|  | (0.01) | (0.01) | (0.01) | (0.01) |
| Education: Bachelor degree | -0.00 | 0.02 | -0.01 | -0.01 |
|  | (0.01) | (0.01) | (0.01) | (0.01) |
| Education: Graduate degree | -0.01 | -0.00 | -0.02* | -0.02* |
|  | (0.01) | (0.01) | (0.01) | (0.01) |
| Income: $30,000 to $60,000 | -0.02** | 0.00 | 0.01 | 0.01 |
|  | (0.01) | (0.01) | (0.01) | (0.01) |
| Income: $60,000 to $90,000 | -0.03** | 0.02 | 0.00 | 0.00 |
|  | (0.01) | (0.01) | (0.01) | (0.01) |
| Income: $90,000 to $120,000 | -0.06*** | 0.01 | 0.01 | 0.01 |
|  | (0.01) | (0.01) | (0.01) | (0.01) |
| Income: $120,000 or more | -0.06*** | 0.01 | -0.01 | -0.01 |
|  | (0.01) | (0.01) | (0.01) | (0.01) |
| Generalized trust | 0.04*** | 0.04*** | 0.04*** | 0.04*** |
|  | (0.01) | (0.01) | (0.01) | (0.01) |
| Supports government spending on poor | 0.16*** | 0.04*** | 0.08*** | 0.08*** |
|  | (0.01) | (0.01) | (0.01) | (0.01) |
| Frequent contact | 0.06*** | 0.04*** | 0.05*** | 0.05*** |
|  | (0.01) | (0.01) | (0.01) | (0.01) |
| Linguistic threat | -0.01 | -0.21*** | -0.05*** | -0.05*** |
|  | (0.01) | (0.01) | (0.01) | (0.01) |
| Economic situation: home | 0.01*** | 0.01** | 0.01*** | 0.01*** |
|  | (0.00) | (0.00) | (0.00) | (0.00) |
| Economic situation: province | 0.01*** | 0.01*** | 0.01*** | 0.01*** |
|  | (0.00) | (0.00) | (0.00) | (0.00) |
| Economic situation: unemployment rate | -0.10 | -0.29* | -0.15 | -0.15 |
|  | (0.14) | (0.14) | (0.13) | (0.13) |
| Proportion: indigenous | -0.47 |  |  |  |
|  | (0.26) |  |  |  |
| Proportion: English speakers |  | 0.11*** |  |  |
|  |  | (0.02) |  |  |
| Proportion: immigrants |  |  | 0.03 |  |
|  |  |  | (0.02) |  |
| Proportion: Arab |  |  |  | 0.03 |
|  |  |  |  | (0.02) |
| National identification | 0.09*** | -0.02 | 0.07*** | 0.07*** |
|  | (0.02) | (0.02) | (0.02) | (0.02) |
| Civic boundaries | 0.07** | 0.05* | 0.07*** | 0.07*** |
|  | (0.02) | (0.02) | (0.02) | (0.02) |
| Ethnic boundaries | -0.14*** | -0.11*** | -0.16*** | -0.16*** |
|  | (0.01) | (0.01) | (0.01) | (0.01) |
| Cultural boundaries | -0.11*** | -0.17*** | -0.06*** | -0.06*** |
|  | (0.02) | (0.02) | (0.01) | (0.01) |
| Num.Obs. | 6,448 | 6,448 | 6,448 | 6,448 |
| *R*^2^_adj._ | 0.15 | 0.18 | 0.17 | 0.28 |

*Note:* * *p* < .05; ** *p* < .01; *** *p* < .001. Contextual data at the municipality (i.e., census subdivision) level, compiled from the 2016 Canadian census. Reference categories for education and income are “high school or less” and “under $30,000,” respectively.

**Table A3**. Logistic (Models 1-3) and OLS (Model 4) regression models showing effects of national identity variables on policy supports, with individual and contextual controls. Standard errors in parentheses, clustered by respondents’ forward sorting area.

|  | (1) | (2) | (3) | (4) |
| --- | --- | --- | --- | --- |
|  | Support for indigenous resource control | Support for English services | Support for decreased immigration | Support for banning religious symbols |
| Constant | -0-83** | 1.84*** | -1.11*** | 0.29*** |
|  | (0.32) | (0.27) | (0.32) | (0.03) |
| Age | -0.00 | 0.01*** | -0.00 | 0.00*** |
|  | (0.00) | (0.00) | (0.00) | (0.00) |
| Female | 0.68*** | -0.20** | -0.04 | -0.09*** |
|  | (0.07) | (0.07) | (0.06) | (0.01) |
| Education: College/CEGEP/Trade school | -0.07 | 0.12 | -0.15 | 0.03** |
|  | (0.09) | (0.06) | (0.08) | (0.01) |
| Education: Some university | 0.24* | 0.44*** | -0.84*** | 0.02 |
|  | (0.11) | (0.10) | (0.12) | (0.01) |
| Education: Bachelors degree | -0.06 | 0.22* | -0.52*** | 0.02 |
|  | (0.10) | (0.09) | (0.10) | (0.01) |
| Education: Graduate degree | -0.08 | 0.24 | -0.45** | -0.04 |
|  | (0.15) | (0.16) | (0.17) | (0.02) |
| Income: $30,000 to $60,000 | -0.36** | -0.04 | -0.43*** | 0.01 |
|  | (0.11) | (0.10) | (0.10) | (0.01) |
| Income: $60,000 to $90,000 | -0.46*** | -0.07 | -0.33*** | 0.02 |
|  | (0.12) | (0.11) | (0.10) | (0.01) |
| Income: $90,000 to $120,000 | -0.59*** | -0.27** | -0.47*** | 0.02 |
|  | (0.13) | (0.10) | (0.11) | (0.01) |
| Income: $120,000 or more | -0.96*** | -0.31** | -0.47*** | 0.01 |
|  | (0.12) | (0.11) | (0.13) | (0.01) |
| Generalized trust | 0.31*** | 0.27** | -0.62*** | -0.03*** |
|  | (0.07) | (0.07) | (0.08) | (0.01) |
| Supports government spending on poor | 1.99*** | 0.90*** | -0.83*** | -0.08** |
|  | (0.11) | (0.11) | (0.13) | (0.01) |
| Frequent contact | 0.36*** | 0.02 | 0.09 | -0.04*** |
|  | (0.09) | (0.08) | (0.08) | (0.01) |
| Linguistic threat | 0.09 | -1.61*** | 1.15*** | 0.12*** |
|  | (0.14) | (0.14) | (0.13) | (0.02) |
| Economic situation: home | 0.06** | 0.01 | -0.06*** | -0.01*** |
|  | (0.01) | (0.01) | (0.02) | (0.00) |
| Economic situation: province | 0.02 | 0.06*** | -0.12*** | 0.00*** |
|  | (0.02) | (0.02) | (0.02) | (0.00) |
| Economic situation: unemployment rate | 5.13** | -0.67 | -0.24 | -0.43* |
|  | (1.77) | (1.39) | (1.83) | (0.19) |
| Proportion: indigenous peoples | -10.00*** |  |  |  |
|  | (2.97) |  |  |  |
| Proportion: English speakers |  | 1.48*** |  |  |
|  |  | (0.24) |  |  |
| Proportion: immigrants |  |  | 0.43 |  |
|  |  |  | (0.25) |  |
| Proportion: Arab |  |  |  | 0.23* |
|  |  |  |  | (0.09) |
| National identification | 0.90*** | -0.10 | 0.07 | 0.08*** |
|  | (0.17) | (0.21) | (0.20) | (0.02) |
| Civic boundaries | -0.06 | 0.67** | -0.28 | 0.11*** |
|  | (0.24) | (0.25) | (0.25) | (0.03) |
| Ethnic boundaries | -0.49*** | -0.41*** | 1.89*** | 0.03** |
|  | (0.11) | (0.09) | (0.12) | (0.01) |
| Cultural boundaries | -0.72** | -1.88*** | 1.59*** | 0.38*** |
|  | (0.23) | (0.22) | (0.25) | (0.03) |
| Num.Obs. | 6,448 | 6,448 | 6,448 | 6,448 |
| AIC | 6398.67 | 7156.37 | 6695.17 |  |
| *R*^2^_adj._ |  |  |  | 0.20 |

*Note:* * *p* < .05; ** *p* < .01; *** *p* < .001. Standard errors in parentheses. Respondents are aggregated into localities based on the first three digits of their postal code. Contextual data at the municipality (i.e., census subdivision) level, compiled from the 2016 Canadian census. Reference categories for education and income are “high school or less” and “under $30,000,” respectively.
